# Mental Health and Work Attitudes among People Resuming Work during the COVID-19 Pandemic: A Cross-Sectional Study in China

**DOI:** 10.3390/ijerph17145059

**Published:** 2020-07-14

**Authors:** Lili Song, Yong Wang, ZhengLin Li, Ying Yang, Hao Li

**Affiliations:** 1 CAS Key Laboratory of Mental Health, Institute of Psychology, Beijing 100101, China; songll@psych.ac.cn; 2 Department of Psychology, University of Chinese Academy of Sciences, Beijing 100049, China; yangying19@psych.ac.cn; 3 School of psychology, Capital Normal University, Beijing 100037, China; 2183502075@cnu.edu.cn; 4 CAS Key Laboratory of Behavioral Science, Institute of Psychology, Beijing 100101, China; 5 Plateau Center of Brain Sciences, Tibet University, Lasa 850000, China; futanghu888@126.com; 6 School of Medicine, Tibet University, Lasa 850000, China

**Keywords:** COVID-19, resume work, mental health, work attitudes, work engagement, job satisfaction, turnover intention, employees

## Abstract

The unprecedented outbreak of the Coronavirus Disease 2019 (COVID-19) caused an economic downturn and increased the unemployment rate in China. In this context, employees face health and social economic stressors. To assess their mental health (i.e., anxiety, depression, insomnia and somatization) and work attitudes (i.e., work engagement, job satisfaction and turnover intention) as well as the associated factors, we conducted a cross-sectional study among people who resumed work after the Spring Festival holiday during the COVID-19 pandemic. The results show that the prevalence of anxiety, depression, insomnia and somatization among these people was 12.7%, 13.5%, 20.7% and 6.6%, respectively. The major risk factor for mental health was worrying about unemployment, and the main protective factors were psychological strengths (i.e., resilience and optimism). Regarding work attitudes, the percentage of people who felt more satisfied with their job (43.8%) was larger than that of those who felt less satisfied (26.9%), while the percentage of people who thought about quitting their job more frequently (15.7%) was smaller than that of those who considered it less frequently (63.2%). However, work engagement was lower than usual. Similar to the factors associated with mental health, the major risk factor for work attitudes was also worrying about unemployment, and the main protective factors were resilience and optimism. In addition, the nature of the organization, job status, age, position and income changes were also related to these work attitudes. Our findings shed light on the need for organization administrators to be aware of the status of and factors associated with employees’ mental health and work attitudes during the COVID-19 pandemic. Policies or interventions could be developed based on our findings.

## 1. Introduction

In December 2019, an unprecedented outbreak of Coronavirus Disease 2019 (COVID-19) occurred in China and soon escalated to a public health emergency on 21 January 2020. To control the COVID-19 outbreak, China locked down Wuhan, a metropolitan area of 12 million people, on 23 January 2020. Subsequently, varying levels of restrictive measures were implemented across China during the Spring Festival, a week-long national holiday that was celebrated this year from 24–30 January. However, by the end of the Spring Festival holiday, the pandemic was still spreading rapidly. To prevent more people from contracting COVID-19, the government extended the holiday to 10 February 2020. Nevertheless, during this period, the pandemic had not been effectively controlled, many people had to resume work at home, and only part of the workforce was allowed to return to the office after seeking approval from the government. Fortunately, by the end of February, because of effective pandemic prevention and control, an increasing number of Chinese workers returned to the office or combined working at home and in the office. In other words, some staff were scheduled to work at home, while others worked at the office in shifts. This flexible approach to the resumption of work after the Spring Festival lasted until late April 2020.

During this period, China’s economy suffered great losses. According to the National Bureau of Statics, China’s GDP in the first quarter dropped by 6.8% compared to that in the same period last year, a 40-year record low [1]. Because of the COVID-19 pandemic, many enterprises stopped work and production, resulting in a decrease in profits. It has been reported that the profits of industrial enterprises above a designated size in China decreased by 36.7% [2]. Subsequently, the unemployment rate reached 6.2% in February, 5.9% in March, and 6.0% in April, approximately one percentage point higher than the unemployment rate in the same period last year [3]. In the context of this economic downturn caused by the COVID-19 pandemic, many working adults are in danger of losing their jobs or having their salaries cut, which may threaten their mental health [4,5]. In addition, their work attitudes may also change accordingly. Some people may be more satisfied with their jobs if their organization has enacted sound policies, while others may be less satisfied if their salary has been cut [6,7,8]. Furthermore, considering the high unemployment rate and economic depression, people’s turnover intention may decrease even if their income decreases. However, as they cannot work or exercise as usual, they may not be as engaged in work as before [9].

To date, most studies on working adults have focused on medical and nursing staff during the COVID-19 pandemic [10,11], and only one study has examined the mental health of people who returned to work in February [12]. Therefore, the status quo of people resuming work after the Spring Festival until April during the COVID-19 pandemic is not well understood, especially their work attitudes. Exploring work attitudes during the COVID-19 pandemic is of great importance, since work attitudes are the antecedents of work and organizational behaviors, which can further determine job performance and organizational effective functioning [13]. Furthermore, knowledge regarding the factors influencing mental health and work attitudes among this population during the COVID-19 pandemic is limited. Economic recovery and improvement in people’s living standards largely depend on these workforces; therefore, it is important to understand the extent of and factors associated with their mental health and work attitudes such that we can identify employees in need and design effective employee assistant programs to prevent mental disorders and develop positive work attitudes. In addition, most research explores the risk factors of people’s status quo under the COVID-19 pandemic, but relatively few studies have examined the protective factors, especially from the psychological resources perspective. As suggested by Kalaitzaki, Tamiolaki and Rovithis, research on the COVID-19 could shift to examining and enhancing the factors that help people cope with the negative effects of the pandemic, and achieve health and well-being while being exposed to the pandemic threat [14]. Therefore, we further introduce psychological strengths in our study to examine their positive effects on these employees during the COVID-19 pandemic.

According to the Conservation of Resources (COR) theory, people strive to obtain, retain, and protect resources, and feel stressed when their resources are lost or threatened with loss [15,16]. During the COVID-19 pandemic, employees have faced various resources losses such as unemployment and salary cuts, which may have resulted in psychological distress [17]. In such circumstances, resource gain becomes particularly important for people to replenish these diminished resources. As psychological strengths, resilience and optimism represent personal characteristic resources that encourage building new resources in adversity for individuals to more easily adapt to changing and demanding circumstances [15,18]. For example, resilience, which refers to “the ability of an individual to withstand adversity” [19] (p. 10), can help employees overcome adverse events and recover quickly [20], thus safeguarding employees’ mental health [18,21], enhancing their job satisfaction [22] and work engagement [23], as well as reducing turnover rate [24]. Optimism, defined as an individual’s general expectation regarding future positive outcomes [25,26], also plays a crucial role when people encounter stressful events. It has been found that optimists tend to use approach coping strategies aiming to eliminate, reduce and manage stressors. They can also adjust their coping strategies to meet the demands of the stressors at hand [25,27]. Therefore, they experience less depression and anxiety, and can maintain healthy mental state [28,29]. In addition, since optimistic people hold positive expectations and believe that current stressful circumstances or negative events can change to become better in the future [30], they are highly motivated and tend to exert more effort in work despite adverse circumstances [26], which can lead to better performance, job satisfaction, and organizational commitment [22]. Hence, we expect these two psychological strengths to help employees build new resources in the stressful and adverse environment caused by the COVID-19 pandemic and, thus, serve as protective factors for employees’ mental health and work attitudes.

When exploring factors associated with mental health, individuals’ sociodemographic factors, such as age, gender, and education is commonly included [31,32,33,34,35]. Generally, the female sex tends to associated with more mental problems, possibly because they are vulnerable to stress [32,34]. For example, a nationwide survey of Chinese people found that females experienced more distress than their male counterparts during the COVID-19 pandemic [32], and similar results were found in in Iran and Brazil [36,37]. Education is also related to people’s mental health. For instance, the nationwide survey mentioned above found that people with higher education tended to experience more distress during the COVID-19 pandemic possibly because they have higher self-awareness of their health conditions [32]. A study conducted in Brazil discovered similar results and further found that younger people experienced higher levels of distress [37].

In addition, employees’ sociodemographic background is also associated with their work attitudes. Some variables such as age, gender, education, tenure and position are typically included when exploring employees’ work attitudes [38]. For example, it has been found that increase in age led to more job satisfaction, probably due to personal-cost calculation [13,39]. Additionally, employees’ work attitudes vary in different types of organizations [13,40]. Generally, employees in public sectors are thought to be more satisfied with their jobs because their work environment is relatively relaxed and they have more secure welfare. However, other evidence indicated that employees in privately owned enterprises had a higher level of job satisfaction than those in state-owned enterprises, because the work in privately owned enterprises is more autonomous and challenging [13]. Nevertheless, since the outbreak of COVID-19, the production and operation of enterprises have been greatly affected. As revealed in a report by China Enterprise Reform and Development Society, the pandemic has the greatest impact on private enterprises, followed by state-owned enterprises and foreign-funded enterprises [41]. Under such circumstances, employees may value stability and avoid risk first. Thus, we expect employees in private enterprises to be less satisfied with their job, become less engaged in their work, and have higher turnover intention. Additionally, work attitudes may also vary among people with different job statuses. For instance, an experimental study found that people working from home had a higher level of job satisfaction [42] and a survey conducted by the videoconferencing company Owl Labs in 2019 found that remote workers were happier, stayed in their jobs longer and worked more hours since they had better work-life balance, less stress, and no commute [43]. During the COVID-19 pandemic, people working at home or in shifts have a lower chance of contracting COVID-19; thus, we hypothesize that these employees will have more positive work attitudes than those working at office.

In summary, in the present study, we first assess the mental health status and work attitudes of people who resumed work after the Spring Festival during the COVID-19 pandemic. Specifically, we examine the prevalence of anxiety, depression, insomnia, and somatization, which are typical indicators of one’s mental health, and work engagement, job satisfaction and turnover intention, which are the major components of work attitudes [8,13,44,45,46,47]. Then, we integrate the changes caused by the economic environment (i.e., unemployment and income change), psychological strengths (i.e., resilience and optimism) and some critical sociodemographic information into our study to investigate the risk and protective factors associated with these working people’s mental health and work attitudes. By doing so, we expect that this study can shed light on the status quo of this population and provide evidence for professionals to facilitate the development of interventions to help people stay mentally healthy and maintain positive work attitudes during the COVID-19 pandemic.

## 2. Materials and Methods 

### 2.1. Participants and Procedure

We conducted a cross-sectional survey from 9–22 April, approximately two and a half months into the COVID-19 emergency in China. The study was approved by the ethics committee of Institute of Psychology, Chinese Academy of Sciences (No. H19031). All participants voluntarily gave their informed consent to participate in the study after being informed about the purpose of the study. The procedures of this study complied with the provisions of the Declaration of Helsinki regarding research on human participants. Regarding the inclusion criteria, all the participants were working adults who were not infected by the virus and began to work at home or at office after the Spring Festival (a week-long national holiday lasting from 24–30 January). By using convenience and snowball sampling, participants were recruited to complete an anonymous online questionnaire that collected data about their sociodemographic information, mental health and work attitudes. To ensure the quality of the data, only one questionnaire could be submitted per IP address, and a simple lie detection question was added in the middle of the questionnaire, i.e., “To judge whether your responses are genuine, please choose ‘strongly disagree’ for this question”. Finally, we collected valid responses from 709 participants. As a reward, all these participants received 6 yuan and a report on their mental health and work attitudes. 

### 2.2. Measurements

Sociodemographic data included sex, age, tenure, education level (i.e., high/vocational school or below, three-year college degree, bachelor’s degree, or postgraduate degree), work position (i.e., ordinary staff, junior manager, middle manager, or senior manager), nature of work organization (i.e., state organ/public institution/social organization, state-owned enterprise, private enterprise or foreign-funded enterprise), and job status (i.e., working at home, working at office, or working alternately at home or office). In addition, participants reported their income changes by answering the question: “After the outbreak of COVID-19, did your income change?” Responses were given on a five-point scale, with “1” representing “decreased a lot”, “2” “decreased”, “3” “no change”, “4” “increased”, and “5” “increased a lot”. Participants were further asked to report the extent to which they were worried that COVID-19 would make them unemployed, from 1 (“not at all”) to 4 (“very much”).

Mental health was measured by assessing anxiety, depression, insomnia, and somatization. Specifically, anxiety was assessed by the 7-item Generalized Anxiety Disorder Scale (GAD-7) [48]. Items were rated for the last two weeks using a four-point scale ranging from 0 (“not at all”) to 3 (“nearly every day”). In this study, we defined a total score ≥9 as indicating the presence of anxiety symptoms [49]. Depression was assessed by the 10-item Chinese short version of the Center for Epidemiologic Studies Depression Scale (CES-D). Items were rated for the last week using a four-point scale ranging from 0 (almost never or not at all) to 3 (most of the time). In the current research, we defined a score ≥14 as indicative of depression [50]. Insomnia was assessed by the 7-item Insomnia Severity Index (ISI) [51]. Items were rated for the last two weeks using a five-point rating scale ranging from 0 (“no problem”) to 3 (“very severe problem”). A total score ≥8 indicated that insomnia was present [52]. Somatic symptoms were measured by the 12-item somatization subscale from the Symptom Check List-90-revised [53,54]. Items were rated on a 5-point Likert scale (from 0 “not at all” to 4 “extremely”). A total score ≥24 indicated the presence of somatic symptoms [55]. In the present study, the Cronbach’s alpha values for the four measures were 0.93, 0.84, 0.90, and 0.84.

Work attitudes were measured by assessing work engagement, job satisfaction and turnover intention. Specifically, work engagement was assessed using the nine-item Utrecht Work Engagement Scale [56]. Items were rated on a 7-point Likert scale (0 = never, 6 = always). In the present study, the Cronbach’s alpha value for this measure was 0.94. Job satisfaction was measured with one item adapted from the overall job satisfaction scale of the Michigan Organizational Assessment Scale [57]. Participants were asked to rate their degree of agreement with the item “Since the outbreak of COVID-19, I am more satisfied with my present job than before” on a five-point scale ranging from 1 (strongly disagree) to 5 (strongly agree). Similarly, turnover intention was measured with one item adapted from the turnover intention scale [58]. Participants were asked to rate item “Since the outbreak of COVID-19, I think about quitting my job more often than before” on a five-point scale ranging from 1 (strongly disagree) to 5 (strongly agree).

Psychological strengths were measured by evaluating resilience and optimism. Specifically, resilience was assessed with the 10-item Connor-Davidson Resilience Scale (CD-RISC-10) [59]. Each item was rated on a 5-point scale from 0 (‘not true at all’) to 4 (‘true nearly all the time’). In the current study, the Cronbach’s alpha value for this measure was 0.95. Optimism was measured by the revised Life Orientation Test (LOT-R) [60]. There were 10 items in this measure, with 4 items serving as fillers. Respondents are asked to rate the extent of their agreement with these items using a 5-point Likert-type scale ranging from 0 (“strongly disagree”) to 4 (“strongly agree”). In the present study, the Cronbach’s alpha value for this measure was 0.70.

### 2.3. Statistical Analyses 

IBM SPSS Statistics 24.0 (IBM, Armonk, NY, USA) was used to analyze the data. First, an analysis of the descriptive statistics was conducted to illustrate the sociodemographic information, mental health, work attitudes and psychological strengths of the participants. Then, multivariate logistic regression analyses were performed using stepwise variable selection, and all variables were entered into the model to explore their independent influence on mental health (i.e., anxiety, depression, insomnia, and somatization). Regarding work attitudes (i.e., work engagement, job satisfaction, and turnover intention), a series of ANOVAs was first conducted to investigate the impact of sex, job status, and organizations, which were non-ordinal categorical variables, on these attitudes. Then, linear regression analyses were performed using stepwise variable selection, and all the variables except sex, organization, and job status were entered into the model to explore their independent influence on work attitudes. All hypotheses were tested at a significance level of 0.05.

## 3. Results

### 3.1. Sociodemographic Descriptions

Our participants were from 26 provinces and municipalities in China. Beijing accounted for the highest percentage (50.1%), followed by Guangdong, at 10.7%. Table 1 presents the characteristics of the participants. During the data collection, 172 (24.3%) participants worked only at home; 362 (51.1%) left home to work at office; and 175 (24.7%) worked alternately at home or office. The participants were from different types of organizations. Specifically, 142 (20.0%) participants worked in state organs/public institution/social organizations; 107 (15.1%) in state-owned enterprises; 331 (46.7%) in private enterprises; and 129 (18.2%) in foreign-funded enterprises. Regarding participants’ income change during the pandemic, although 436 (61.5%) participants reported no change in income, 244 (34.4%) reported that their incomes decreased, and only 39 (4.1%) reported an increase. χ^2^ tests further showed that the income changes varied by organization (χ^2^ = 68.19, *p* < 0.001). Specifically, private enterprises accounted for the largest percentage of people reporting an income decrease, at 47.4%, followed by state-owned enterprises (29.9%), state organs/public institutions/social organizations (22.5%) and foreign-funded enterprises (17.9%). In terms of the extent to which participants were worried that COVID-19 would make them unemployed, 251 (35.4%) participants reported worrying “a little bit” to “very much”.

### 3.2. Mental Health and Its Associated Factors

As shown in Table 1, the prevalence of anxiety, depression, insomnia, and somatization among these working adults was 12.7%, 13.5%, 20.7% and 6.6%, respectively. The multivariate logistic regression analyses (Table 2) showed that four variables were independently associated with anxiety: having a high education level (OR, 1.57; 95% CI, 1.08–2.27; *p* = 0.016) and worrying about unemployment (OR, 2.12; 95% CI, 1.58–2.84; *p* < 0.001) were risk factors for anxiety, while resilience (OR, 0.52; 95% CI, 0.35–0.75; *p* = 0.001) and optimism (OR, 0.27; 95% CI, 0.15–0.47; *p* < 0.01) were protective factors for anxiety. In the depression models, younger age (OR, 0.93; 95% CI, 0.89–0.97; *p* < 0.001) and worrying about unemployment (OR, 1.93; 95% CI, 1.44–2.59; *p* < 0.001) were associated with increased levels of depression, while resilience (OR, 0.36; 95% CI, 0.24–0.53; *p* < 0.001) and optimism (OR, 0.31; 95% CI, 0.18–0.54; *p* < 0.01) were significantly associated with decreased levels of depression. Regarding insomnia, four variables were independently associated with insomnia: sex (OR, 0.57; 95% CI, 0.38–0.86; *p* = 0.008), tenure (OR, 0.95; 95% CI, 0.92–0.98; *p* = 0.004), worrying about unemployment (OR, 1.43; 95% CI, 1.12–1.82; *p* < 0.001) and optimism (OR, 0.47; 95% CI, 0.31–0.72; *p* < 0.01). In terms of somatization, three variables were independently associated with somatization. Specifically, compared with working at the office, working alternately at home or office could reduce the risk of somatization (OR, 0.32; 95% CI, 0.12–0.87; *p* = 0.025); resilience (OR, 0.58; 95% CI, 0.38–0.89; *p* = 0.012) and optimism (OR, 0.25; 95% CI, 0.13–0.49; *p* < 0.01) could also serve as protective factors for somatization.

### 3.3. Work Attitudes and Their Associated Factors

As shown in Table 1, a total of 310 (43.8%) participants reported that they were more satisfied with their present job since the outbreak of COVID-19, while 191 (26.9%) felt less satisfied than before. In terms of turnover intention, only 112 (15.7%) participants reported that their turnover intention was more frequent than before, while with a larger percentage (63.2%) disagreed (including strongly disagreed). Next, we conducted a series of ANOVAs to investigate the impact of sex, job status, and organization, which are non-ordinal categorical variables, on these attitudes (i.e., work engagement, job satisfaction, and turnover intention). The results showed that sex was a significant predictor only of job satisfaction. After the outbreak of COVID-19, females (*M* = 3.29, *SD* = 1.10) were more satisfied with their jobs than males (*M* = 3.07, *SD* = 1.11), *F* (1, 707) = 5.48, *p* = 0.019. In addition, as shown in Table 3, work engagement, job satisfaction, and turnover intention significantly differed by the organization type. Specifically, the participants in private enterprises had the highest level of turnover intention and lowest level of job satisfaction, while people in state organs/public institutions/social organizations and foreign-funded enterprises had higher levels of work engagement and job satisfaction but lower turnover intention. 

Work attitudes also differ among participants with different job statuses. As shown in Table 4, the participants who worked at home and those who worked alternately at home or office had higher levels of work engagement than those who worked at office. Similarly, participants working at home and working alternately at home or office were more satisfied with their current jobs than those working at office. There are no differences in turnover intention based on job status.

Linear regression analyses were further performed using stepwise variable selection, and all variables, except for sex, organization and job status, were entered into the model to explore their independent influence on the three aspects of work attitudes. As shown in Table 5, four variables were independently associated with work engagement: age, position, resilience and optimism. They all positively predicted work engagement. In the job satisfaction models, six variables were independently associated with work engagement: age, income change, resilience and optimism positively predicted job satisfaction, while position and worrying about unemployment negatively predicted job satisfaction. In terms of turnover intention, four variables were independently associated with turnover intention: age, income change, and worrying about unemployment negatively predicted turnover intention, while optimism positively predicted turnover intention.

## 4. Discussion

This study examined the mental health and work attitudes of working adults who started to work after the Spring Festival while the COVID-19 pandemic was still spreading. The results showed that the prevalence of anxiety, depression, insomnia, and somatization among these people was 12.7%, 13.5%, 20.7% and 6.6%, respectively. A previous study that also examined anxiety and depression with the same measures in the Chinese public found that the prevalence of anxiety and depression among enterprise or institution workers was 34.8% and 20.1%, respectively [49], much higher percentages than ours. The possible reason may be that their study was conducted from February 3 to 17, 2020, a period during which the number of people infected with COVID-19 was increasing rapidly. Therefore, people in this environment may have felt more anxious and depressed. However, another study, which examined mental health among people returning to work from 24 to 25 February, 2020, reported a much lower prevalence of anxiety and depression than ours, i.e., 3.8% and 3.7%, respectively [12]. There may be two reasons for this. On the one hand, these authors assessed the immediate mental health of employees who just returned to work at office from February 24 to 25. At that time, the economic and unemployment stress caused by the pandemic had not been fully shown. Hence, these employees’ mental health may not have been greatly affected. On the other hand, this discrepancy may be due to the different measurement instruments used. The prevalence of insomnia (21.7%) was also higher than that in Tan et al.’s study (14.5%) [12], but lower than that in non-medical health workers (30.5%) [10]. Regarding somatization, the prevalence (6.6%) was higher than that in Zhang et al.’s study (0.4%) [10]; however, most of our participants’ somatization symptoms were mild (6.1%).

In terms of the factors associated with employees’ mental health, it was found that worrying about unemployment was a major risk factor for anxiety, depression and insomnia. Indeed, jobs are an important part of life for working adults. Being unemployed means that they will lose their source of income and have no security, which may make them anxious and depressed and cause them to have low sleep quality. In addition, some other sociodemographic factors are associated with employees’ mental health during the COVID-19 pandemic. For example, employees with higher education levels were more prone to be anxious and younger employees were more likely to be depressed. These results were consistent with previous studies which revealed that being younger and more educated are risk factors for mental health during the COVID-19 pandemic [32,37]. However, it was found that male employees had higher levels of insomnia than their female counterparts, which seems to counter our general perception that females tend to experience more insomnia symptoms than males. A possible explanation is that the males are generally regarded as the head of the family, and they may assume more responsibilities and feel more pressure under the economic downturn caused by COVID-19, thus leading to relatively poorer mental health. Indeed, recent studies conducted during the COVID-19 pandemic also found similar results indicating that males experienced higher levels of stress [31] and had a higher tendency to develop psychological problems and PTSD [35]. Despite these risk factors associated with employee’s mental health, we found that some protective factors, i.e., resilience and optimism, can prevent these symptoms of mental disorders. That is, people who are resilient and optimistic are more likely to withstand the negative effects of exposure to the COVID-19 pandemic and maintain mentally healthy in the face of adversity. 

This study also examined people’ work attitudes when they resumed work after the Spring Festival. Generally, after the outbreak of COVID-19, the percentage of people who were more satisfied with their job (43.8%) was larger than that of those less satisfied (26.9%), while the percentage of people thinking about quitting their job more frequently (15.7%) was smaller than that of those who considered it less frequently (63.2%). It is possible that people feel contented that they are still employed during the COVID-19 pandemic, and quitting their job may introduce risk into their lives. However, compared with the results reported in previous studies that were not conducted during the COVID-19 pandemic [61,62,63], work engagement in our study was lower, which, to some extent, reflects the negative impact of the COVID-19 pandemic on people’s work engagement. 

In addition, people’s work attitudes varied by organization. For example, employees in private enterprises were the least satisfied with their current job and had the highest turnover intention. This may be due to the fact that private enterprises suffered most during this pandemic [41], which was also reflected in our results suggesting that employees in private enterprises had the highest rate of income decrease. In contrast, state organs/public institutions/social organizations are non-profitable and foreign-funded enterprises have relatively stronger anti-risk ability. Therefore, these two types of organizations tended to be less influenced by the pandemic [41], which was also reflected in our results indicating that employees in these organizations underwent less rate of income decrease compared with other types of organization. Thus, people in state organs/public institutions/social organizations and foreign-funded enterprises comparatively have higher levels of job satisfaction and work engagement and lower levels of turnover intention. Furthermore, work attitudes also vary among people with different job statuses. Specifically, compared with people who went out to work at office, those working at home and working alternately at home or office were more satisfied with their job and engaged in their work, which is consistent with previous findings during non-pandemic times [42,43]. Indeed, when they do not need to go into the office, people can spend time working that would have otherwise been spent commuting. In addition, their risk of contracting COVID-19 decreases because they have contact with fewer people. Therefore, they feel safer and become more satisfied with their job, since their job allows them to work at home.

In this study, we also found several factors related to work attitudes. Generally, older workers were more satisfied with their job, engaged in work and had lower turnover intention. This finding is consistent with a prior study that revealed older workers were less likely to change their work attitudes in the face of adversity since they are more experienced in employing coping strategies to stay engaged in work [9]. However, for people with higher positions, although they were more engaged in their work, they felt less satisfied with their job. It is possible that people in high positions have more duties, especially during the pandemic. Therefore, they must put in more effort at work, and the overload may make them feel less satisfied with their current job. Additionally, a decrease in income and worry about unemployment are risk factors for people’s work attitudes. Specifically, people whose income decreased during the COVID-19 pandemic or who worried about unemployment reported less job satisfaction and more turnover intention. Similar to the factors related to mental health, resilience and optimism are protective factors for work attitudes. These two psychological strengths can help people make positive attributions to present and future and adopt adaptive coping styles when facing the difficulties and challenges posed by the COVID-19 pandemic. Thus, working adults with high levels of resilience and optimism can stay mentally healthy and maintain good attitudes towards work [21,24,26,28,29,64,65].

Our study contributes to the COVID-19 research literature in the following ways. On the one hand, we present the status quo of people who are working during the COVID-19 pandemic, including their mental health and work attitudes. Although previous studies have already investigated some aspects of mental health among working adults [12,66], work attitudes have not been examined. Exploring work attitudes is critical to understand the work status and occupational psychology of people working during the COVID-19 pandemic. On the other hand, we integrate the changes caused by the economic environment (i.e., unemployment and income change) and psychological strengths (resilience and optimism) into our study to investigate the factors associated with people’s mental health and work attitudes. We found that worrying about unemployment was a major risk factor for both mental health and work attitudes, while resilience and optimism served as protective factors. Regarding work attitudes, the nature of the organization, job status, age, position and income changes also play important roles.

Our findings also have practical implications for practitioners. For example, governments can strengthen the implementation of employment security policy. Additionally, organizations can provide support to employees and create a safe atmosphere to reduce employees’ concern about unemployment. Notably, organizations need to be careful about reducing employees’ salaries, since such a decision may have negative effects on employees’ work attitudes, which in turn could affect organizational functioning. As COVID-19 is still spreading globally, organizations in some countries with severe outbreaks should not rush to have employees return to the office to work. If conditions permit, employees can work at home or in shifts, which will increase their job satisfaction and work engagement. In addition, our findings suggest that older employees may employ coping strategies to reduce depression and be more positive at work even during the COVID-19 pandemic. Therefore, managers and supervisors can leverage the existing age-diverse workforce to communicate experiences. For example, they can create opportunities for older employees to share their experiences with their younger colleagues who might be experiencing major public health events for the first time. Finally, resilience and optimism training can be integrated into intervention programs to help people stay mentally healthy and develop positive work attitudes during this special period.

Despite these implications stated above, our study has several limitations which need to be addressed in the future. First, this study was cross-sectional; thus, causal conclusions should be drawn carefully. Future research can use longitudinal designs to investigate the possible causal relationships and the long-term impact of the pandemic on these employees. Furthermore, it could be interesting to explore how employees’ work attitudes are converted into observable behaviors such as actual turnover and job performance. Second, all the data collected were self-reported by the participants, and more objective data could be used in future similar research. Finally, our sample was not nationally representative. Although we attempted to collect data from across the country, the sampling was not proportional to the population. Thus, future research could adopt more accurate sampling to improve the representativeness of the sample and the generalizability of the results.

## 5. Conclusions

Our findings shed light on the need for organization administrators to be aware of the status of and factors associated with mental health and work attitudes among people returning to work after the Spring Festival during the COVID-19 pandemic. Generally, their mental health has been affected to some extent, and their work attitudes changed after the COVID-19 outbreak: employees tended to be more satisfied with their current jobs and had less turnover intention. However, they were not so engaged in their work as before. Furthermore, our results show that worrying about unemployment is a major risk factor for both mental health (i.e., anxiety, depression, insomnia and somatization) and work attitudes (i.e., work engagement, job satisfaction, and turnover intention), while psychological strengths such as resilience and optimism are the main protective factors. The nature of the organization, job status, age, position and income changes also play important roles in work attitudes.

## Figures and Tables

**Table 1:** Characteristics of the participants (*N* = 709).

Variables	Count or Mean (SD)	Percentage
**Sex**		
Male	183	25.8%
Female	526	74.2%
**Age (years) Mean (SD)**	35.35 (6.61)	
**Tenure (years) Mean (SD)**	12.53 (7.20)	
**Education level**		
High/vocational school or below	20	2.8%
Three-year college degree	62	8.7%
Bachelor’s degree	371	52.3%
Postgraduate degree	256	36.1%
**Job status**		
Worked at home	172	24.3%
Worked at office	362	51.1%
Worked alternately at home or office	175	24.7%
**Position**		
Ordinary staff	302	42.6%
Junior manager	162	22.8%
Middle manager	190	26.8%
Senior manger	55	7.8%
**Organization**		
State organ/public institution/social organization	142	20.0%
State-owned enterprise	107	15.1%
Private enterprise	331	46.7%
Foreign-funded enterprise	129	18.2%
**Income change**		
Decreased a lot	74	10.4%
Decreased	170	24.0%
No change	436	61.5%
Increased	25	3.5%
Increased a lot	4	0.6%
**Worry about unemployment caused by COVID-19**		
Not at all	458	64.6%
A little bit	192	27.1%
Moderate	43	6.1%
Very much	16	2.3%
**Anxiety**		
Yes	100	14.1%
No	609	85.9%
**Depression**		
Yes	96	13.5%
No	613	86.5%
**Insomnia**		
Yes	147	20.7%
No	562	79.3%
**Somatization**		
Yes	47	6.6%
No	662	93.4%
**More satisfied with the present job**		
Strongly disagree	45	6.3%
Disagree	146	20.6%
Neutral	208	29.3%
Agree	221	31.2%
Strongly agree	89	12.6%
**More frequent turnover intention**		
Strongly disagree	306	43.2%
Disagree	184	20.0%
Neutral	107	15.1%
Agree	84	11.8%
Strongly agree	28	3.9%
Work engagement	3.42(1.12)	
Optimism	2.75(0.52)	
Resilience	2.92(0.68)	

**Table 2:** Factors associated with mental health.

Variables	OR (95% CI)	*p*
**Models for anxiety**		
Education	1.57 (1.10, 2.24)	0.013
Worry about unemployment	2.12 (1.59, 2.82)	<0.001
Resilience	0.51 (0.35, 0.73)	<0.001
Optimism	0.28 (0.16, 0.48)	<0.001
**Models for depression**		
Age	0.93 (0.89, 0.97)	<0.001
Worry about unemployment	1.93 (1.44, 2.59)	<0.001
Resilience	0.36 (0.24, 0.53)	<0.001
Optimism	0.31 (0.18, 0.54)	<0.001
**Models for insomnia**		
Sex (male vs. female)	0.57 (0.38, 0.86)	0.008
Tenure	0.95 (0.92, 0.98)	0.004
Worry about unemployment	1.43 (1.12, 1.82)	0.005
Optimism	0.47 (0.31, 0.72)	0.001
**Models for somatization**		
Job status (working at office is the default category)		
Working at home	0.67 (0.31, 1.44)	0.307
Working alternately at home and office	0.32 (0.12, 0.87)	0.025
Resilience	0.58 (0.38, 0.89)	0.012
Optimism	0.25 (0.13, 0.49)	<0.001

Note. OR, odds ratio; CI, confidence interval.

**Table 3:** Differences in work attitudes by organization.

	1	2	3	4		
	State Organ/Public Institution/Social Organization	State-Owned Enterprise	Private Enterprise	Foreign-Funded Enterprise	*F*	Post Hoc test
Work engagement	3.46 (0.09)	3.18 (0.11)	3.37 (0.06)	3.68 (0.10)	4.33 **	1>2; 4>2; 4>3
Job satisfaction	3.51 (1.08)	3.21 (1.11)	3.01 (1.11)	3.50 (1.01)	10.31 ***	1>2; 1>3; 4>2; 4>3
Turnover intention	1.63 (0.10)	2.10 (0.11)	2.36 (0.06)	1.81 (0.10)	15.78 ***	3>2>1; 3>4

Note. Standard deviations are in parentheses; ** *p* < 0.01, *** *p* < 0.001.

**Table 4:** Differences in work attitudes by job status.

	1	2	3		
	Worked at Home	Worked at Office	Worked Alternately at Home and Office	*F*	Post Hoc test
Work engagement	3.54 (0.09)	3.31 (0.06)	3.52 (0.08)	3.48 *	1>2; 3>2
Job satisfaction	3.31 (1.15)	3.12 (1.10)	3.39 (1.06)	4.20 *	1>2; 3>2
Turnover intention	1.97 (0.09)	2.12 (0.06)	2.07 (0.09)	0.97	

Note. Standard deviations are in parentheses; * *p* < 0.05.

**Table 5:** Factors associated with work attitudes.

Variables	β (95%CI)	*p*
**Models for work engagement**		
Age	0.08 (0.03, 0.001)	0.033
Position	0.12 (0.05, 0.21)	0.001
Resilience	0.41 (0.56, 0.80)	<0.001
Optimism	0.10 (0.06, 0.37)	0.008
**Models for job satisfaction**		
Age	0.12 (0.73, 0.42)	0.005
Position	−0.10 (−0.20, −0.03)	0.012
Income change	0.08 (0.03, 0.24)	0.029
Worry about unemployment	−0.10 (−0.20, −0.02)	0.015
Resilience	0.15 (0.10, 0.37)	0.001
Optimism	0.32 (0.07, 0.42)	0.005
**Models for turnover intention**		
Age	−0.15 (−0.04, −0.01)	<0.001
Income change	−0.08 (−0.25, −0.01)	0.034
Worry about unemployment	0.20 (1.15, 0.33)	<0.001
Optimism	−0.15 (−0.51, −0.18)	<0.001

## References

[1] National Bureau of Statics The Impact of the Epidemic on the Economy in the First Quarter. http://www.stats.gov.cn/tjsj/zxfb/202004/t20200419_1739666.html.

[2] National Bureau of Statics The Profit of Industrial Enterprises above Designated Size in China Decreased by 36.7% from January to March 2020. http://www.stats.gov.cn/tjsj/zxfb/202004/t20200427_1741735.html.

[3] National Bureau of Statics The Press Conference from National Bureau of Statistics on Chinese Economy in April 2020. http://www.stats.gov.cn/tjsj/sjjd/202005/t20200515_1745719.html.

[4] Loureiro A., Santana P., Nunes C., Almendra R. (2019). The Role of Individual and Neighborhood Characteristics on Mental Health after a Period of Economic Crisis in the Lisbon Region (Portugal): A Multilevel Analysis. Int. J. Environ. Res. Public Health.

[5] Wahlbeck K., Mcdaid D. (2012). Actions to alleviate the mental health impact of the economic crisis. World Psychiatry.

[6] Ravid O., Malul M., Zultan R. (2017). The effect of economic cycles on job satisfaction in a two−sector economy. J. Econ. Behav. Organ..

[7] Solberg I.B., Tomasson K., Aasland O., Tyssen R. (2014). Cross−national comparison of job satisfaction in doctors during economic recession. Occup. Med..

[8] Giannikis S., Nikandrou I. (2013). The impact of corporate entrepreneurship and high−performance work systems on employees’ job attitudes: Empirical evidence from Greece during the economic downturn. Int. J. Hum. Resour. Manage..

[9] Matz-Costa C., Pitt-Catsouphes M., Besen E., Lynch K. (2009). The Difference a Downturn can Make: Assessing the Early Effects of the Economic Crisis on the Employment Experiences of Workers.

[10] Zhang W.R., Wang K., Yin L., Zhao W.F., Xue Q., Peng M., Min B.Q., Tian Q., Leng H.X., Du J.L. (2020). Mental Health and Psychosocial Problems of Medical Health Workers during the COVID-19 Epidemic in China. Psychother. Psychosom..

[11] Shi Y., Wang J., Yang Y., Wang Z., Wang G., Hashimoto K., Zhang K., Liu H. (2020). Knowledge and attitudes of medical staff in Chinese psychiatric hospitals regarding COVID-19. Brain Behav. Immun. Health.

[12] Tan W., Hao F., McIntyre R.S., Jiang L., Jiang X., Zhang L., Zhao X., Zou Y., Hu Y., Luo X. (2020). Is returning to work during the COVID-19 pandemic stressful? A study on immediate mental health status and psychoneuroimmunity prevention measures of Chinese workforce. Brain Behav. Immun..

[13] Wang X. (2008). Analyzing work attitudes of Chinese employees. Chin. Manag. Stud..

[14] Kalaitzaki A.E., Tamiolaki A., Rovithis M. (2020). The healthcare professionals amidst COVID-19 pandemic: A perspective of resilience and posttraumatic growth. Asian J. Psychiatr..

[15] Hobfoll S.E. (2002). Social and Psychological Resources and Adaptation. Rev. Gen. Psychol..

[16] Hobfoll S.E. (1989). Conservation of resources: A new attempt at conceptualizing stress. Am. Psychol..

[17] Freedy J.R., Saladin M.E., Kilpatrick D.G., Resnick H.S., Saunders B.E. (1994). Understanding acute psychological distress following natural disaster. J. Trauma. Stress.

[18] Boudrias J.-S., Desrumaux P., Gaudreau P., Nelson K., Brunet L., Savoie A. (2011). Modeling the experience of psychological health at work: The role of personal resources, social-organizational resources, and job demands. Int. J. Stress Manag..

[19] Jackson D., Firtko A., Edenborough M. (2007). Personal resilience as a strategy for surviving and thriving in the face of workplace adversity: A literature review. J. Adv. Nurs..

[20] Luthans F., Avey J.B., Avolio B.J., Peterson S.J. (2010). The development and resulting performance impact of positive psychological capital. Hum. Resour. Dev. Q..

[21] Vanhove A.J., Herian M.N., Perez A.L.U., Harms P.D., Lester P.B. (2016). Can resilience be developed at work? A meta-analytic review of resilience-building programme effectiveness. J. Occup. Organ. Psychol..

[22] Youssef C.M., Luthans F. (2007). Positive Organizational Behavior in the Workplace. J. Manag..

[23] Gupta M., Shaheen M., Reddy P.K. (2017). Impact of psychological capital on organizational citizenship behavior. J. Manag. Dev..

[24] Shin J., Taylor M.S., Seo M.-G. (2012). Resources for Change: The Relationships of Organizational Inducements and Psychological Resilience to Employees’ Attitudes and Behaviors toward Organizational Change. Acad. Manag. J..

[25] Carver C.S., Scheier M.F., Segerstrom S.C. (2010). Optimism. Clin. Psychol. Rev..

[26] Carver C.S., Scheier M.F. (2014). Dispositional optimism. Trends Cogn. Sci..

[27] Nes L.S., Segerstrom S.C. (2006). Dispositional Optimism and Coping: A Meta-Analytic Review. Pers. Soc. Psychol. Rev..

[28] Alarcon G.M., Bowling N.A., Khazon S. (2013). Great expectations: A meta-analytic examination of optimism and hope. Pers. Individ. Dif..

[29] Andersson G. (1996). The benefits of optimism: A meta-analytic review of the life orientation test. Pers. Individ. Dif..

[30] Chang E.C., Yu E.A., Lee J.Y., Hirsch J.K., Kupfermann Y., Kahle E.R. (2012). An Examination of Optimism/Pessimism and Suicide Risk in Primary Care Patients: Does Belief in a Changeable Future Make a Difference?. Cognit. Ther. Res..

[31] Wang C., Pan R., Wan X., Tan Y., Xu L., Ho C.S., Ho R.C. (2020). Immediate Psychological Responses and Associated Factors during the Initial Stage of the 2019 Coronavirus Disease (COVID-19) Epidemic among the General Population in China. Int. J. Environ. Res. Public Health.

[32] Qiu J., Shen B., Zhao M., Wang Z., Xie B., Xu Y. (2020). A nationwide survey of psychological distress among Chinese people in the COVID-19 epidemic: Implications and policy recommendations. Gen. Psychiatr..

[33] Zhang J., Lu H., Zeng H., Zhang S., Du Q., Jiang T., Du B. (2020). The differential psychological distress of populations affected by the COVID-19 pandemic. Brain Behav. Immun..

[34] Sareen J., Erickson J., Medved M.I., Asmundson G.J., Enns M.W., Stein M., Leslie W., Doupe M., Logsetty S. (2013). Risk factors for post-injury mental health problems. Depress. Anxiety.

[35] Liang L., Ren H., Cao R., Hu Y., Mei S. (2020). The Effect of COVID-19 on Youth Mental Health. Psychiatr. Q..

[36] Jahanshahi A.A., Dinani M.M., Madavani A.N., Li J., Zhang S.X. (2020). The distress of Iranian adults during the Covid-19 pandemic—More distressed than the Chinese and with different predictors. Brain Behav. Immun..

[37] Zhang S.X., Wang Y., Jahanshahi A.A., Jia J., Schmitt V.G.H. (2020). First study on mental distress in Brazil during the COVID-19 crisis. medRxiv.

[38] Bernerth J.B., Aguinis H. (2016). A Critical Review and Best-Practice Recommendations for Control Variable Usage. Pers. Psychol..

[39] Ross C.E., Reskin B.F. (1992). Education, Control at Work, and Job Satisfaction. Soc. Sci. Res..

[40] Chiu W.C.K. (2002). Do types of economic ownership matter in getting employees to commit? An exploratory study in the People’s Republic of China. Int. J. Hum. Resour. Manag..

[41] China Enterprise Reform and Development Society Self-Employed Households and Private Enterprises are most Seriously Affected: A Report on the Impact of the Epidemic. https://baijiahao.baidu.com/s?id=1662470208526646749&wfr=spider&for=pc.

[42] Bloom N., Liang J., Roberts J., Ying Z.J. (2015). Does Working from Home Work? Evidence from a Chinese Experiment. Q. J. Econ..

[43] Owl Labs State of Remote Work 2019. https://www.owllabs.com/state-of-remote-work/2019?hs_preview=jWDXIXgj-13385250578.

[44] Avolio B.J., Gardner W.L., Walumbwa F.O., Luthans F., May D.R. (2004). Unlocking the mask: A look at the process by which authentic leaders impact follower attitudes and behaviors. Leadersh. Q..

[45] Avey J.B., Reichard R.J., Luthans F., Mhatre K.H. (2011). Meta-analysis of the impact of positive psychological capital on employee attitudes, behaviors, and performance. Hum. Resour. Dev. Q..

[46] Luthans F., Zhu W., Avolio B.J. (2006). The impact of efficacy on work attitudes across cultures. J. World Bus..

[47] Avey J.B., Wernsing T.S., Luthans F. (2008). Can Positive Employees Help Positive Organizational Change? Impact of Psychological Capital and Emotions on Relevant Attitudes and Behaviors. J. Appl. Behav. Sci..

[48] Spitzer R.L., Kroenke K., Williams J.B., Lowe B. (2006). A brief measure for assessing generalized anxiety disorder: The GAD-7. Arch. Intern. Med..

[49] Huang Y., Zhao N. (2020). Generalized anxiety disorder, depressive symptoms and sleep quality during COVID-19 outbreak in China: A web-based cross-sectional survey. Psychiatry Res..

[50] Wang J., Zhou Y., Liang Y., Liu Z. (2019). A Large Sample Survey of Tibetan People on the Qinghai-Tibet Plateau: Current Situation of Depression and Risk Factors. Int. J. Environ. Res. Public Health.

[51] Morin C.M., Belleville G., Bélanger L., Ivers H. (2011). The Insomnia Severity Index: Psychometric Indicators to Detect Insomnia Cases and Evaluate Treatment Response. Sleep.

[52] Wong Y.S., Zhang D.X., Li C.K., Yip H.K., Chan C.C., Ling Y.M., Lo S.L., Woo M.S., Sun Y.Y., Ma H. (2017). Comparing the Effects of Mindfulness-Based Cognitive Therapy and Sleep Psycho-Education with Exercise on Chronic Insomnia: A Randomised Controlled Trial. Psychother. Psychosom..

[53] Wang Z. (1984). Symptom Check List (SCL-90). Shanghai Jingshen Yixue.

[54] Chen S., Li L. (2003). Re-testing reliability, validity, and norm applicability of SCL-90. Chin. J. Nerv. Ment. Dis..

[55] Chen X., Li P., Wang F., Ji G., Miao L. (2017). Psychological Results of 438 Patients with persisting Gastroesophageal Reflux Disease Symptoms by Symptom Checklist 90-Revised Questionnaire. Euroasian J. Hepatogastroenterol..

[56] Schaufeli W.B., Bakker A.B., Salanova M. (2006). The Measurement of Work Engagement With a Short Questionnaire A Cross-National Study. Educ. Psychol. Meas..

[57] Cammann C., Fichman M., Jenkins D., Klesh J. (1979). The Michigan Organizational Assessment Questionnaire.

[58] Mobley W.H., Horner S.O., Hollingsworth A.T. (1978). An evaluation of precursors of hospital employee turnover. J. Appl. Psychol..

[59] Wang L., Shi Z., Zhang Y., Zhang Z. (2010). Psychometric properties of the 10-item Connor-Davidson Resilience Scale in Chinese earthquake victims. Psychiatry Clin. Neurosci..

[60] Scheier M.F., Carver C.S., Bridges M.W. (1994). Distinguishing optimism from neuroticism (and trait anxiety, self−mastery, and self-esteem): A reevaluation of the Life Orientation Test. JPSP.

[61] Leroy H., Anseel F., Dimitrova N.G., Sels L. (2013). Mindfulness, authentic functioning, and work engagement: A growth modeling approach. J. Vocat. Behav..

[62] Meyers M.C., Kooij D., Kroon B., de Reuver R., van Woerkom M. (2020). Organizational Support for Strengths Use, Work Engagement, and Contextual Performance: The Moderating Role of Age. Appl. Res. Qual. Life.

[63] Sousa M., Dierendonck D.V. (2017). Servant Leadership and the Effect of the Interaction Between Humility, Action, and Hierarchical Power on Follower Engagement. J. Bus. Ethics.

[64] Duffy R.D., Bott E.M., Allan B.A., Torrey C.L. (2013). Examining a model of life satisfaction among unemployed adults. J. Couns. Psychol..

[65] King D.D., Newman A., Luthans F. (2016). Not if, but when we need resilience in the workplace. J. Organ. Behav..

[66] Zhang S.X., Wang Y., Rauch A., Wei F. (2020). Unprecedented disruption of lives and work: Health, distress and life satisfaction of working adults in China one month into the COVID-19 outbreak. Psychiatry Res..

